# The Potential Therapeutic Agent Mepacrine Protects Caco-2 Cells against *Clostridium perfringens* Enterotoxin Action

**DOI:** 10.1128/mSphere.00352-17

**Published:** 2017-08-30

**Authors:** John C. Freedman, Matthew R. Hendricks, Bruce A. McClane

**Affiliations:** Department of Microbiology and Molecular Genetics, University of Pittsburgh School of Medicine, Pittsburgh, Pennsylvania, USA; University of Maryland Medical Center

**Keywords:** *Clostridium perfringens*, enterotoxin, mepacrine, pore-forming toxin, quinacrine

## Abstract

*Clostridium perfringens* enterotoxin (CPE) causes the diarrhea associated with a common bacterial food poisoning and many antibiotic-associated diarrhea cases. The severity of some CPE-mediated disease cases warrants the development of potential therapeutics. A previous study showed that the presence of mepacrine inhibited CPE-induced electrophysiology effects in artificial lipid bilayers lacking CPE receptors. However, that study did not assess whether mepacrine inactivates CPE or, instead, inhibits a step in CPE action. Furthermore, CPE action in host cells is complex, involving the toxin binding to receptors, receptor-bound CPE oligomerizing into a prepore on the membrane surface, and β-hairpins in the CPE prepore inserting into the membrane to form a pore that induces cell death. Therefore, the current study evaluated the ability of mepacrine to protect cells from CPE. This drug was found to reduce CPE-induced cytotoxicity in Caco-2 cells. This protection did not involve mepacrine inactivation of CPE, indicating that mepacrine affects one or more steps in CPE action. Western blotting then demonstrated that mepacrine decreases CPE pore levels in Caco-2 cells. This mepacrine-induced reduction in CPE pore levels did not involve CPE binding inhibition but rather an increase in CPE monomer dissociation due to mepacrine interactions with Caco-2 membranes. In addition, mepacrine was also shown to inhibit CPE pores when already present in Caco-2 cells. These *in vitro* studies, which identified two mepacrine-sensitive steps in CPE-induced cytotoxicity, add support to further testing of the therapeutic potential of mepacrine against CPE-mediated disease.

**IMPORTANCE** *Clostridium perfringens* enterotoxin (CPE) causes the gastrointestinal (GI) symptoms of a common bacterial food poisoning and several nonfoodborne human GI diseases. A previous study showed that, via an undetermined mechanism, the presence of mepacrine blocks CPE-induced electrophysiologic activity in artificial membranes. The current study now demonstrates that mepacrine also inhibits CPE-induced cytotoxicity in human enterocyte-like Caco-2 cells and that mepacrine does not directly inactivate CPE. Instead, this drug reduces both CPE pore formation and CPE pore activity in Caco-2 cells. These results suggest mepacrine as a therapeutic candidate for treating CPE-mediated GI diseases.

## Abstract (precis)

*Clostridium perfringens* enterotoxin (CPE) causes the gastrointestinal (GI) symptoms of a common bacterial food poisoning and several nonfoodborne human GI diseases. A previous study showed that, via an undetermined mechanism, the presence of mepacrine blocks CPE-induced electrophysiologic activity in artificial membranes. The current study now demonstrates that mepacrine also inhibits CPE-induced cytotoxicity in human enterocyte-like Caco-2 cells and that mepacrine does not directly inactivate CPE. Instead, this drug reduces both CPE pore formation and CPE pore activity in Caco-2 cells. These results suggest mepacrine as a therapeutic candidate for treating CPE-mediated GI diseases.

## INTRODUCTION

*Clostridium perfringens* type A strains producing the *C. perfringens* enterotoxin (CPE) are the second most common causative agent of bacterial food poisoning (1). In the United States alone, about 1 million cases of this foodborne disease occur annually (2). *C. perfringens* type A food poisoning usually involves diarrhea and abdominal cramps that self-resolve within 24 h (1). Death, while uncommon, does occur in elderly or debilitated people (3). Additionally, two exceptionally severe outbreaks of this food poisoning caused fatalities in several relatively young and healthy individuals who had preexisting, severe, drug-induced constipation or fecal impaction (4, 5). Besides their role in food poisoning, CPE-producing type A strains are also the causative agent for ∼10% of all antibiotic-associated diarrhea (AAD) cases (6). CPE-associated AAD is usually more severe, and has a longer duration (up to several weeks), than *C. perfringens* type A food poisoning (6). Studies fulfilling molecular Koch’s postulates demonstrated that CPE production is essential for the enteric pathogenicity of strains causing either *C. perfringens* type A food poisoning or CPE-associated AAD (7).

CPE is a 35 kDa single-polypeptide, pore-forming toxin that has a unique primary amino acid sequence but belongs structurally to the aerolysin toxin family (8, 9). This enterotoxin is produced when *C. perfringens* sporulates in the intestine, and it is then released upon lysis of the mother cell (1). The C-terminal receptor-binding domain of CPE binds to claudin receptors to form an ∼90-kDa “small complex” comprised of the enterotoxin, receptor claudins and, possibly, nonreceptor claudins (10–13). Using its N-terminal domain, claudin-bound CPE then oligomerizes to produce a hexameric prepore on the membrane surface (11, 14). In this prepore, β-hairpin loops extend from the N-terminal domain of CPE to form a β-barrel that inserts into the lipid bilayer of plasma membranes to create a pore (15). The CPE oligomeric pore, known as the CH-1 large complex, is cation permeable and allows for a Ca^2+^ influx into cells, which results in CPE-induced cell death (16–20).

Therapeutic modalities would be useful to attenuate CPE effects during severe cases of food poisoning or CPE-induced chronic diarrhea from AAD. Toward that goal, two synthetic peptide-based inhibitors have been previously explored as inhibitors of CPE activity. A peptide corresponding to the 30 C-terminal amino acids of CPE was shown to successfully block CPE binding to rabbit small intestinal brush border membranes by competing against the native enterotoxin for claudin binding (21). Similarly, a peptide corresponding to the second extracellular loop sequence of claudin-4 was successfully used as a receptor decoy to protect Caco-2 cells or rabbit small intestine from CPE effects (22, 23). However, those peptide-based approaches required the use of high concentrations of expensive synthetic peptides that are susceptible to inactivation in the gastrointestinal tract, limiting their practicality as potential clinical therapeutics.

An alternative approach was suggested by a study reporting that the presence of mepacrine reduces CPE-induced electrophysiologic activity in pure synthetic lipid bilayers (24). Mepacrine, an ∼400-Da acridine derivative (25), is administered orally and has already been used clinically to prevent and/or treat protozoal infections (25), including intestinal infections caused by *Giardia* spp. (26, 27). The successful clinical use of mepacrine to treat other intestinal infections, coupled with the reported ability of mepacrine to inhibit CPE-induced electrophysiologic activity in artificial membranes, provides justification for further analysis of this drug as a potential CPE therapeutic.

However, the initial study (24) that detected mepacrine inhibition of CPE-induced electrophysiologic activity did not determine whether the drug directly inactivated CPE or, instead, interfered with CPE action by reducing the toxin’s binding, pore formation, or pore activity. Also, that previous study used a noncellular model system (artificial membranes) lacking CPE receptors, making it necessary to evaluate the effects of mepacrine on CPE-induced cytotoxicity against host cells. This is important because, (i) the presence of receptors is essential for CPE-induced cytotoxicity at physiologically relevant CPE concentrations (28, 29), (ii) in cells, these receptors remain associated with the CPE pore (11) and thus could affect the mepacrine sensitivity of CPE pores, and (iii) host cell responses to CPE are more complicated than those of artificial membranes, e.g., the presence of mepacrine could affect the ability of CPE-treated host cells to release membrane vesicles containing CPE pores (30) or to degrade CPE pores.

Therefore, the goal of the current study was to test whether mepacrine protects intact host cells against CPE-induced cytotoxicity and, if so, to identify whether this protection involves CPE inactivation or interference with one or more steps in CPE action. Using a human enterocyte-like Caco-2 cell culture model, mepacrine was shown to protect against CPE-induced cytotoxicity, and two mechanisms for this protection were identified.

## RESULTS

### Mepacrine protects Caco-2 cells from CPE-induced cytotoxicity.

**FIG 1:**
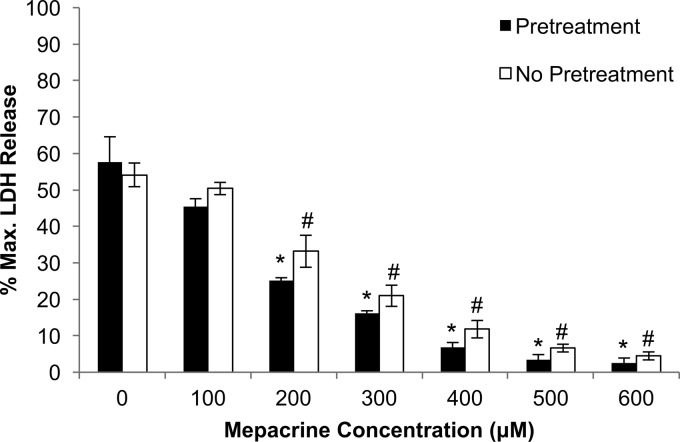
Mepacrine protects Caco-2 cells from CPE-induced cytotoxicity. Caco-2 cells were preincubated for 1 h with HBSS containing mepacrine at the indicated concentrations. Following the 1 h pretreatment, CPE was added to the Caco-2 cells with the indicated mepacrine concentrations and the cells were then incubated at 37°C for an additional 1 h. A parallel experiment was performed in which Caco-2 cells were treated simultaneously with CPE and mepacrine, but without any mepacrine pretreatment. At this time, cytotoxicity was measured (as the percent maximal LDH release; *y* axis). The error bars represent standard errors (*n* = 3), and one-way ANOVA was performed to compare each treatment with results with Caco-2 cells not treated with mepacrine. *, *P* < 0.05 for mepacrine-pretreated cells; #, *P* < 0.05 for nonpretreated cells.

Since the presence of mepacrine reportedly reduces CPE-induced electrophysiologic activity in artificial membranes (24), the current study first evaluated whether the presence of mepacrine also interferes with the development of CPE-mediated cytotoxicity in Caco-2 cells. In an initial experiment, Caco-2 cells were pretreated with mepacrine at 0 to 600 μM (Fig. 1) for 30 min at 37°C, followed by a 60-min challenge at 37°C with Hanks’ balanced salt solution (HBSS) containing both 1 μg/ml of CPE (2.6 nM) and the equivalent dose of mepacrine as used during the pretreatment. Results of this experiment indicated that, in a dose-dependent manner, the presence of mepacrine before and during toxin treatment protected Caco-2 cells from CPE-induced cell death. Significant protection was evident at 200 μM mepacrine, and virtually full protection was achieved using a 500 μM concentration of mepacrine.

A second experiment (Fig. 1) then evaluated whether mepacrine pretreatment is necessary to achieve protection of Caco-2 cells against CPE-induced cytotoxicity. In this additional experiment, Caco-2 cells were directly treated with both CPE and mepacrine (no mepacrine pretreatment). Significant protection was again observed using mepacrine concentrations higher than 200 μM, and essentially complete protection was achieved using between 500 and 600 μM mepacrine.

**FIG 2:**
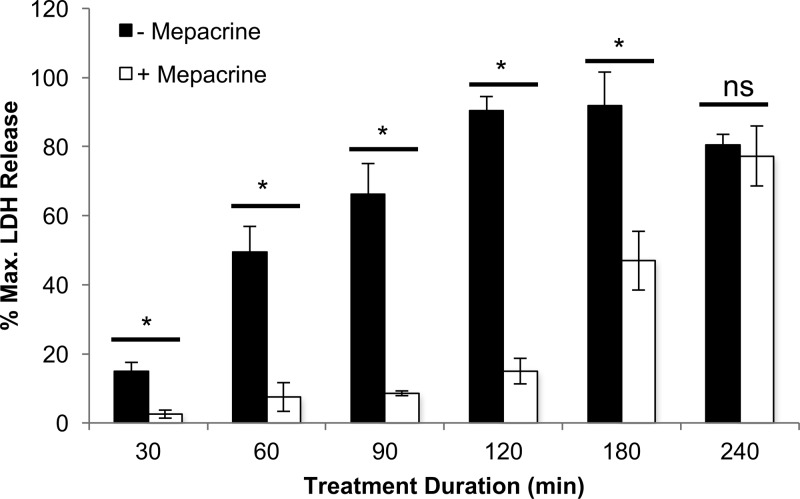
Mepacrine protection of Caco-2 cells at different treatment times. Caco-2 cells were treated simultaneously with HBSS or mepacrine (600 μM) plus CPE for the indicated times (*x* axis) at 37°C. Following incubation, the cytotoxicity was measured based on LDH release (*y* axis). The error bars represent standard errors (*n* = 3), and Student’s unpaired *t* test was performed to compare the cytotoxicity of CPE-treated Caco-2 cells with versus without mepacrine. *, *P* < 0.05; ns, not significant.

The next experiment assessed the duration of mepacrine protection against CPE-induced cytotoxicity (Fig. 2). When Caco-2 cell viability was measured after treatment of those cells with HBSS containing 1 μg/ml of CPE in the presence or absence of 600 μM mepacrine, the presence of mepacrine provided virtually complete protection against CPE-induced cytotoxicity at 30, 60, 90, and 120 min. Even at 180 min, mepacrine still provided partial, but statistically significant, protection to the Caco-2 cells from CPE. However, those protective effects ceased by 240 min.

**FIG 3:**
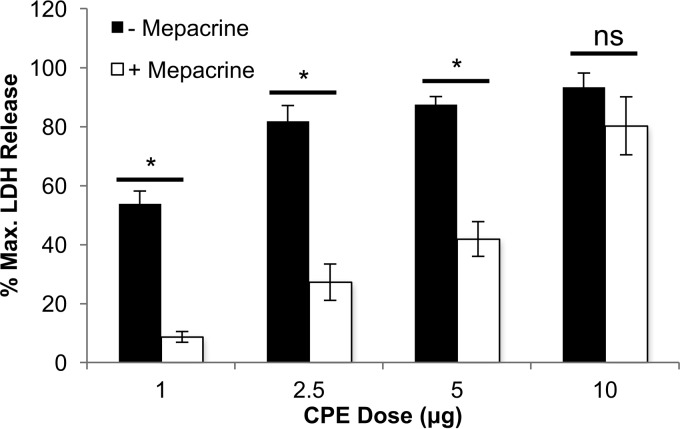
Mepacrine protection of Caco-2 cells at increasing CPE doses. Caco-2 cells were treated simultaneously with HBSS or mepacrine (600 μM) plus CPE at the indicated toxin doses (*x* axis) for 1 h at 37°C. Following incubation, the cytotoxicity was measured based on LDH release (*y* axis). The error bars represent standard errors (*n* = 3), and Student’s unpaired *t* test was performed to compare the cytotoxicity of CPE-treated Caco-2 cells with versus without mepacrine. *, *P* < 0.05; ns, not significant.

To investigate whether there is a maximum CPE concentration for which mepacrine is protective, Caco-2 cells were treated for 60 min at 37°C with HBSS that did or did not contain 600 μM mepacrine, along with CPE concentrations of 1, 2.5, 5, or 10 μg/ml (Fig. 3). As in the experiment shown in Fig. 1, Caco-2 cells were completely protected by mepacrine when challenged with 1 μg/ml of CPE. Mepacrine also significantly protected Caco-2 cells from 2.5 or 5 μg/ml concentrations of CPE. However, significant protection ceased when Caco-2 cells were treated with 10 μg/ml of CPE (Fig. 3).

Taken together, these initial experiments demonstrated that (i) mepacrine can prevent CPE-induced Caco-2 cell death, (ii) mepacrine exerts these inhibitory effects on CPE for up to 180 min, and (iii) mepacrine is protective against CPE even at moderately high enterotoxin doses.

### Mepacrine exposure does not irreversibly inactivate CPE.

A possible explanation for the results shown in Fig. 1 to 3 is that exposure to mepacrine inactivates the enterotoxin. To test this possibility, CPE was incubated with mepacrine (600 μM) for 1 h and then dialyzed overnight to remove free mepacrine. This treatment did not reduce CPE-induced cytotoxicity (data not shown) relative to toxin that had been similarly incubated and dialyzed in the absence of mepacrine.

To confirm this result, CPE was incubated for 1 h with mepacrine (600 μM) and then analyzed over a PD-10 chromatography column to remove free mepacrine. The chromatographed toxin still retained the same cytotoxic activity as an equivalent amount of CPE that had been incubated for 1 h without mepacrine and then analyzed over a PD-10 chromatography column (data not shown). Additionally, mepacrine (which can be detected by fluorescence at 488 nm) was not detected in the eluate of the PD-10 column containing CPE (data not shown), indicating that mepacrine likely does not interact and inactivate free, monomeric CPE.

### Mepacrine affects CH-1 levels in Caco-2 cells.

**FIG 4:**
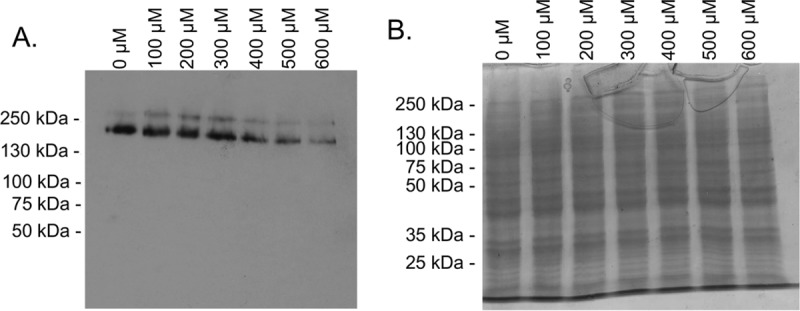
Mepacrine decreases CPE large complex levels in Caco-2 cells. Caco-2 cells were treated simultaneously with CPE and mepacrine at the indicated concentrations for 1 h at 37°C. Following incubation, cells were collected and lysed, and total proteins were separated by SDS-PAGE. Following electrophoresis, lysates were transferred to nitrocellulose and CPE large complex was detected using Western blotting with anti-CPE antisera (A). This blot displays a representative result of 3 experiments. (B) A duplicate gel was loaded and stained with Coomassie blue as a loading control.

Since the dialysis and PD-10 experiments showed that the mechanism of mepacrine protection of Caco-2 cells from CPE-induced cytotoxicity does not involve toxin inactivation, this drug must interfere with one or more steps in CPE action. CPE kills sensitive cells via a series of events, i.e., CPE binding to claudins, CPE oligomerization into a prepore, and pore formation and activity following insertion of β-hairpin loops to form a beta-barrel (31). The CH-1 pore complex is required for CPE to cause Caco-2 cell cytotoxicity (14), so we performed an experiment that examined whether mepacrine affects CH-1 levels in Caco-2 cells. When Caco-2 cells were treated simultaneously with CPE and increasing mepacrine concentrations (0 to 600 μM) for 60 min at 37°C, CPE Western blotting detected a mepacrine concentration-dependent reduction in the amount of CH-1 complex present in Caco-2 cells (Fig. 4A). A second SDS-PAGE gel was loaded with the same aliquot volume of cell lysates as for the gel used for CPE Western blotting in Fig. 4A and then stained with Coomassie blue to demonstrate equal loading of samples (Fig. 4B). Collectively, these results revealed that, at least in part, higher concentrations of mepacrine can protect Caco-2 cells against CPE-induced cytotoxicity by reducing CH-1 pore levels in these cells.

### Mepacrine effects on CPE binding to Caco-2 cells.

**FIG 5:**
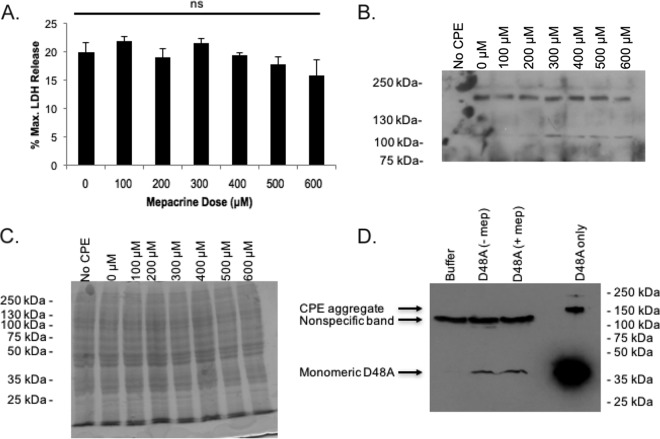
Mepacrine does not affect CPE binding to Caco-2 cells. Caco-2 cells were treated simultaneously with CPE and mepacrine at the indicated concentrations for 1 h at 4°C. Following three washes to remove unbound CPE, prewarmed HBSS was added and cells were incubated for 1 h at 37°C. (A) At this time, cellular cytotoxicity was measured, and a one-way ANOVA was performed to compare each treatment. ns, not significant. (B and C) Western blotting was performed to compare levels of CPE large complex in the treated cells (B), and a duplicate gel was run and then stained with Coomassie blue as a loading control (C). (D) A site-directed CPE variant incapable of oligomerization and insertion (rCPE_D48A_) was added to Caco-2 cells simultaneously with (+ mep) or without (− mep) mepacrine and incubated for 30 min at 37°C. Lysates were then processed for Western blotting with anti-CPE antisera to compare levels of rCPE_D48A_ binding (arrow). The blots in panels B and D are representative of 3 independent experiments. Note the ∼120-kDa immunoreactive band in Caco-2 cells for panel B is background and not due to CPE, since it was also present in Caco-2 cells treated with buffer alone. The larger band in the rCPE_D48A_ only (no cells) lane of panel D is due to nonspecific aggregation of CPE at high concentrations during SDS-PAGE, as described previously (18).

Inhibition of either binding or postbinding steps in CPE action could explain the mepacrine-induced decrease in CH-1 pore levels detected in Caco-2 cells (Fig. 4). To determine if the presence of lower CH-1 complex levels observed in the presence of mepacrine is due to reduced CPE binding to Caco-2 cells, two experiments were performed. First, CPE was bound to host cells in the presence or absence of mepacrine at 4°C, a temperature at which CPE binding occurs but further steps in CPE action, i.e., CPE oligomerization and toxin insertion to form pores and cell death, are inhibited (11, 32). When Caco-2 cells were treated at 4°C in the presence of increasing concentrations of mepacrine (0 to 600 μM), washed with cold HBSS to remove unbound CPE, and then treated with warm (37°C) HBSS, cell death was similar at all mepacrine concentrations tested (Fig. 5A). Besides providing further support for our conclusion that mepacrine exposure does not inactivate CPE, this result strongly suggested that CPE binding levels are equivalent in the presence or absence of mepacrine. This conclusion was supported by CPE Western blotting, which detected the presence of equal amounts of CPE (mainly in the form of CH-1) in all cells, i.e., the presence of mepacrine during CPE treatment at 4°C did not inhibit binding of the enterotoxin to its receptors (Fig. 5B). As in Fig. 4, an identical, but Coomassie-stained, SDS-PAGE gel was used as a loading control (Fig. 5C).

A second experiment then directly demonstrated that mepacrine does not inhibit CPE binding, even at 37°C. For this purpose, the binding at 37°C of rCPE_D48A_, a noncytotoxic CPE point variant that binds to, but cannot oligomerize in (14), Caco-2 cells was compared in the presence versus absence of 600 μM mepacrine. CPE Western blotting results (Fig. 5D) revealed that mepacrine did not inhibit rCPE_D48A_ binding to Caco-2 cells at 37°C.

### Mepacrine inhibits a postbinding step of CPE action.

**FIG 6:**
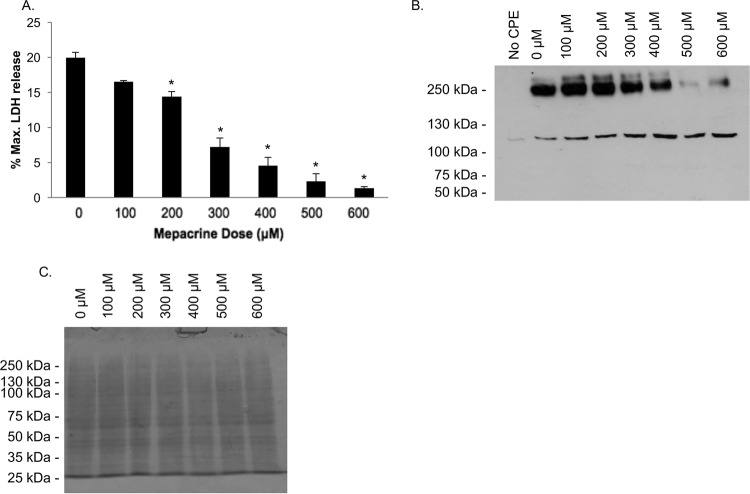
Mepacrine decreases CPE pore complex levels at a postbinding step. CPE was incubated with Caco-2 cells for 30 min at 4°C to allow for binding but not CPE CH-1 formation. After three washes, warm HBSS was added for 60 min to the CPE-treated cells along with the indicated concentration of mepacrine. (A) Cellular cytotoxicity was measured, and a one-way ANOVA was performed to compare each treatment. *, *P* < 0.05. (B and C) Western blotting was performed to compare levels of CPE large complex in the treated cells (B), and a duplicate gel was run and stained with Coomassie blue as a loading control (C). The Western blot in panel B shows a representative result of 3 experiments.

Since mepacrine did not appreciably affect CPE binding to Caco-2 cells, an experiment was performed to evaluate directly whether the observed mepacrine-mediated decrease in CPE-induced cytotoxicity results from an inhibition of postbinding steps in the CPE mechanism of action. For this study, CPE was bound (in the absence of mepacrine) to Caco-2 cells at 4°C before washing and incubation in warm HBSS containing increasing concentrations of mepacrine (0 to 600 μM). After a 60-min treatment at 37°C, the amount of cytotoxicity progressively decreased in the presence of increasing mepacrine concentrations (Fig. 6A).

To support the conclusion that mepacrine inhibits a postbinding step in CPE activity, CH-1 complex levels in those cells were examined (Fig. 6B). Western blotting revealed that, despite equal amounts of CPE bound at 4°C at the start of the experiment, the subsequent addition of mepacrine decreased CH-1 pore levels in these cells, consistent with mepacrine affecting CPE action at a postbinding step. A loading control was performed, as described above (Fig. 6C).

### Mepacrine affects CH-1 formation by interacting with Caco-2 cells.

**FIG 7:**
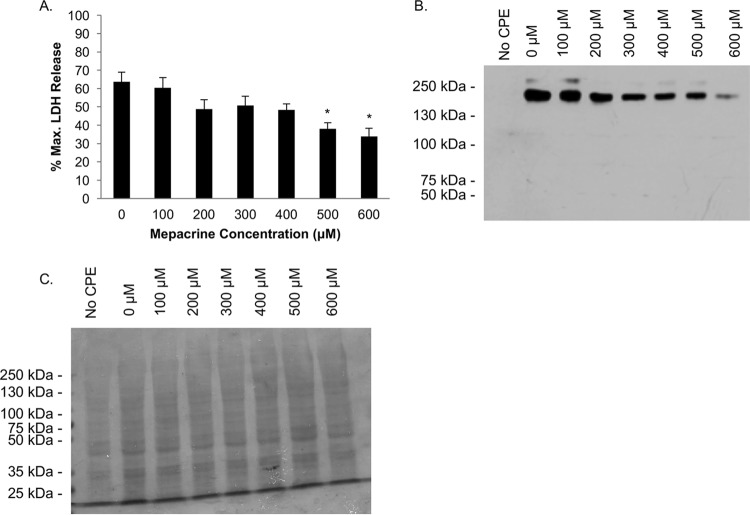
Caco-2 cell pretreatment with mepacrine protects Caco-2 cells from CPE**.** Caco-2 cells were preincubated with mepacrine for 30 min at 37°C. Following the removal of mepacrine and 3 subsequent washes, the cells were incubated with CPE for 1 h at 37°C. Following this incubation, Caco-2 cell cytotoxicity (*, *P* < 0.05, one-way ANOVA) (A) and CH-1 complex formation were assessed by Western blotting for CPE (B). A duplicate Coomassie-stained gel was used to assess loading (C). The blot in panel B shows a representative result of 3 experiments.

Mepacrine is known to interact with and modify eukaryotic cellular membranes (33–35). Since our results shown in Fig. 5 and 6 indicated that mepacrine affects postbinding events in CPE action, mepacrine might inhibit membrane-dependent steps in CPE action, e.g., by reducing toxin oligomerization or insertion into membranes. To test this hypothesis, Caco-2 cells were pretreated at 37°C with increasing concentrations of mepacrine for 30 min. When the culture supernatant was then removed, the cells were washed 3 times, and CPE in warm HBSS was then added to these Caco-2 cells. A cytotoxicity assay revealed that preincubation with 600 μM mepacrine significantly decreased CPE-induced cytotoxicity (Fig. 7A). When the amount of CH-1 pore complex present in these Caco-2 cells was measured by Western blotting (Fig. 7B), pretreatment at 37°C with >200 μM mepacrine decreased CH-1 levels. Results with a loading control are shown in Fig. 7C. Taken together, these cytotoxicity and pore complex formation results indicated that mepacrine interactions with membranes can impact CH-1 pore levels and cytotoxicity in CPE-treated cells.

### Mepacrine promotes CPE monomer dissociation from the Caco-2 cell surface.

**FIG 8:**
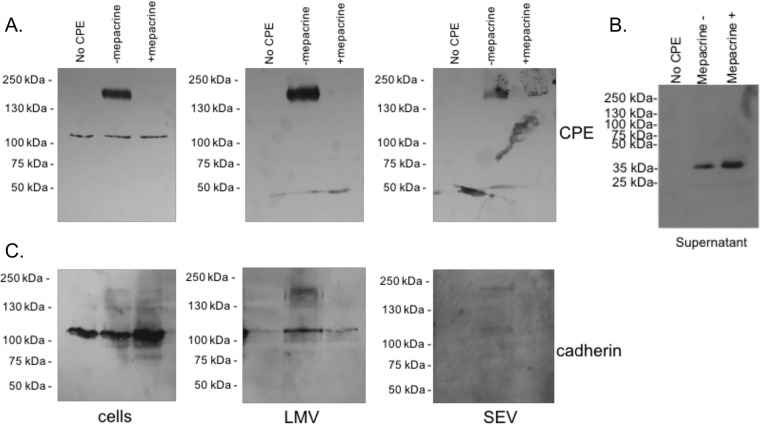
Mepacrine inhibits pore formation by bound CPE. CPE was bound to Caco-2 cells for 30 min at 4°C. Cells were washed three times and subsequently treated with (+) or without (-) mepacrine for 1 h at 37°C. (A and B) Western blotting for CPE was performed to assess CPE CH-1 pore formation in Caco-2 cells (A, left panel) and to identify CPE species present in LMVs (A, middle panel), SEVs (A, right panel), and supernatants (B). (C) Western blotting for pan-cadherin was performed to assess relative vesicle release under each treatment condition (cells [left panel], LMVs [middle panel], and SEVs [right panel]). The results show representative images of at least 3 experimental trials.

Because our results (shown in Fig. 7) indicated that mepacrine inhibits postbinding steps, at least in part via its interaction with Caco-2 cell membranes, it was hypothesized that mepacrine might inhibit CPE-induced cytotoxicity by inducing the release of CPE-containing membrane vesicles or CPE dissociation from Caco-2 cells. To test these postulates, Caco-2 cells were incubated with CPE at 4°C in the absence of mepacrine to allow toxin binding and then, after washing, those cells were incubated in warm HBSS with or without 600 μM mepacrine for 60 min. Differential centrifugation of the Caco-2 cell culture supernatants was then performed, including an initial 10,000 × *g* centrifugation for 30 min to remove large membrane vesicles (LMVs). The LMV-depleted supernatants were then centrifuged further at 100,000 × *g* for 90 min to remove small extracellular vesicles (SEVs). As observed in earlier experiments described here, the amount of CPE complex decreased in cells treated with mepacrine (Fig. 8A, left panel). When each centrifugation fraction was screened for CH-1 complex or CPE monomer by CPE Western blotting, the presence of mepacrine decreased the amount of CH-1 complex present in LMVs (Fig. 8A, middle panel) and in SEVs (Fig. 8A, right panel). Therefore, mepacrine did not increase the release of CPE-containing vesicles from Caco-2 cells. This was confirmed by Western blotting using an anti-pan-cadherin antibody (Fig. 8B). When these cells were treated with CPE only, an increase in cadherin signal associated with LMVs or SEVs was observed relative to untreated Caco-2 cells, indicating an increase in LMV release (Fig. 8B, middle panel) and SEV release (Fig. 8B, right panel). However, when cells were treated with both CPE and mepacrine, a decreased amount of cadherin staining was observed relative to cells treated with CPE only, strongly suggesting that mepacrine decreased CPE-induced vesicle release (Fig. 8B).

Additional Western blotting experiments then demonstrated that mepacrine treatment of Caco-2 cells did cause an increase in the levels of CPE present in the supernatant fractions from those centrifugations (Fig. 8C). Using ImageJ densitometric analysis of 3 separate Western blots, this increase was determined to be ∼2-fold higher (i.e., a 90% ± 20% increase [mean ± standard error of the mean]) for free CPE in supernatants in the presence of mepacrine. Notably, this dissociated CPE was in the form of free monomer. The results shown in Fig. 8C support a mepacine-induced decrease in CPE pores in Caco-2 cells that resulted from increased dissociation of bound toxin monomer prior to pore formation.

### Mepacrine also blocks CPE pore activity.

**FIG 9:**
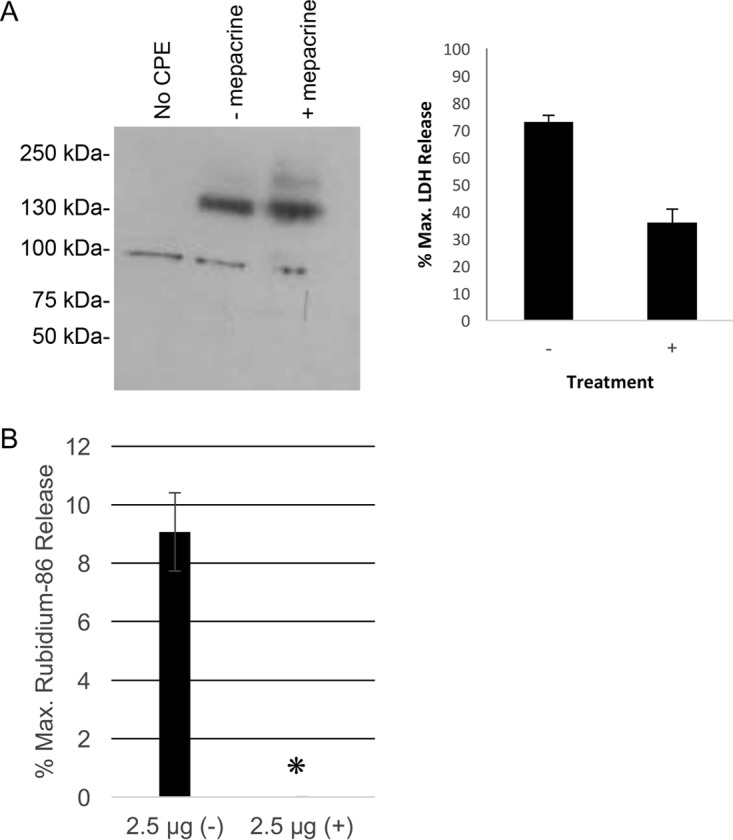
Mepacrine blocks the activity of the CPE pore. (A) CPE was incubated with Caco-2 cells for 10 min at 37°C to allow for equal amounts of CPE pore formation. CPE was then removed, and cells were washed 3 times. HBSS only (-) or mepacrine (+) was added to the cells and the mixtures were incubated for 1 h at 37°C. (A) Western blotting was employed to confirm equal amounts of CPE CH-1 complex (left panel), and cytotoxicity was measured (right). *, *P* < 0.05, Student’s *t* test). The analyses in panel A were performed 3 times. (B) ^86^Rb release assay results with (+) and without (-) mepacrine present (*n* = 3).

The results presented above did not exclude the possibility that mepacrine might exert a second postbinding inhibitory effect on CPE activity in addition to promoting CPE dissociation. To address if mepacrine also blocks CPE pore activity in intact host cells, Caco-2 cells were first pulsed with CPE for 10 min at 37°C to allow for CH-1 pore formation to occur in membranes. When the cells were washed 3 times to remove unbound CPE and then treated with either HBSS or HBSS plus 600 μM mepacrine for 60 min at 37°C, CH-1 pore levels were unaffected by the presence of mepacrine (Fig. 9A, left panel). This result was consistent with the results shown in Fig. 8, indicating that mepacrine affects CH-1 pore levels by increasing CPE monomer dissociation prior to pore formation rather than by causing a loss of the CPE pore complex. However, despite containing the same levels of CH-1 pore complex, cells treated with mepacrine displayed significantly reduced amounts of cytotoxicity (Fig. 9A, right panel), strongly suggesting that mepacrine inhibits CPE pore activity in these cells.

The conclusion that mepacrine can block the CPE pore was then confirmed using an ^86^Rb release assay that is commonly used to assess CPE pore activity (15, 36, 37). For this experiment, Caco-2 cells were briefly (10 min) pretreated with CPE at 37°C to form pores, washed, and then treated with HBSS that did or did not contain 600 μM mepacrine. The results obtained (Fig. 9C) demonstrated that the presence of mepacrine nearly eliminated CPE-induced ^86^Rb release.

## DISCUSSION

As mentioned in our introduction, the availability of CPE-directed therapeutics could be helpful for ameliorating several CPE-associated medical conditions. A previous study had suggested that the drug mepacrine might be a candidate CPE therapeutic because the presence of this drug interferes with CPE-induced electrophysiologic activity in artificial lipid bilayers (24). However, that study did not distinguish whether mepacrine inactivates the CPE protein or instead interferes with some step in CPE action, i.e., whether this drug affects CPE binding, CPE pore formation, or CPE pore activity. Furthermore, it was specifically important to determine whether mepacrine is not only protective against CPE electrophysiologic activity in artificial membranes but also inhibits CPE-induced cytotoxicity in mammalian cells, where receptors are present and complex phenomena like membrane vesicle release occur (30).

Therefore, a first major contribution of the current study entailed demonstrating that mepacrine protects Caco-2 cells, which are enterocyte-like human cells, against CPE-induced cytotoxicity. This drug provided durable (>3 h) protection against CPE doses commonly encountered during food poisoning, as estimated in enzyme-linked immunosorbent assay analyses of stool samples from patients with CPE-associated diarrhea (28). While the current work provides proof-of-principle results, it should be appreciated that mepacrine derivatives or related drugs in the mepacrine family may offer even more efficient protection against CPE, possibly including protection against even higher doses of this enterotoxin.

As mentioned, mepacrine can be administered orally and has proven clinical efficacy against other intestinal infectious diseases (26, 27). This argues positively for the potential use of mepacrine against CPE-mediated disease, although confirmation by future *in vivo* testing is required. In that regard, it is notable that significant protection against CPE-induced cytotoxicity was achieved in the current study using >200 μM concentrations of mepacrine. Those concentrations lie within the mepacrine dose range used clinically to treat *Giardia* infections (38). It is also notable that mepacrine can be absorbed from the intestines (39), opening the possibility that this drug is also effective in ameliorating CPE-induced enterotoxemias where CPE is thought to be absorbed from the intestines and then damages internal organs, like the liver (40).

A second major contribution of the current study was our evaluation of how mepacrine protects cells against CPE. The current study showed that this protection does not involve mepacrine inactivation of the CPE protein. Instead, it was determined that mepacrine interferes with some, but not all, steps in CPE’s action. Initial studies demonstrated that mepacrine does not affect CPE binding to Caco-2 cells. Neither the binding of CPE at 4°C nor the binding at 37°C of the rCPE_D48A_ variant, which binds normally but cannot oligomerize (14), was affected by the presence of this drug.

The ability of mepacrine to affect one or more postbinding steps in CPE action was then confirmed by demonstrating that mepacrine inhibited CPE cytotoxic activity even when CPE was bound at 4°C before the addition of this drug. When mechanistic studies of this postbinding protective effect were pursued, a strong decrease in cell-associated CPE pore levels was detected. This effect was not due to mepacrine treatment enhancing the release of pore-containing vesicles from CPE-treated Caco-2 cells. Instead, a substantial increase in the dissociation of bound CPE monomer was observed in mepacrine-treated cells. Because it was shown previously that the oligomerized CPE prepore remains intact even after dissociation (11, 41), the increase in dissociation of the CPE monomer induced by mepacrine strongly suggests that mepacrine can alter membranes to inhibit CPE oligomerization.

Further evidence that exposure to mepacrine alters membranes to inhibit CPE pore formation included the observation that a brief preincubation of cells with mepacrine offered protection against subsequent CPE challenge. The nature of those membrane modifications induced by mepacrine exposure is likely to be complicated. Mepacrine has been shown previously to exert numerous effects on membranes, including modifying membrane lipids and inhibiting phospholipase activity (33, 34). Also consistent with the conclusion that mepacrine effects on CPE pore levels occur prior to pore formation is the observed stability of the CPE pore once formed in membranes (Fig. 9), despite the presence of mepacrine.

The current work then identified a second step in CPE-induced cytotoxicity that is sensitive to mepacrine. Addition of mepacrine inhibited CPE pore activity in host cells. Importantly, mepacrine even affected CPE pore activity in Caco-2 cells when added after those pores had formed in membranes of the host cells. This observation further supports the therapeutic potential of this drug for treating CPE-mediated disease, since any therapeutic would likely be administered after some pores are already present. Furthermore, the ability of 200 to 300 μM mepacrine concentrations to significantly inhibit CPE-induced cytotoxicity (Fig. 1) while not substantially affecting CH-1 pore levels (Fig. 4) suggests that inhibition of pore activity has predominant importance for protecting cells against CPE action at lower mepacrine concentrations.

The mechanism by which mepacrine blocks CPE pore activity is unknown. However, it is notable that mepacrine also blocks the activity of several mammalian membrane channels, including the nicotinic acetylcholine receptor and the GABA receptor-gated Cl^−^ channel (42, 43), as well as inhibiting the cytoplasmic entry of several intracellularly active bacteria toxins, including the *Clostridium botulinum* C2 toxin and *Bacillus anthracis* lethal toxin, by inhibiting the pores formed by these toxins in endosomal membranes (44). This broad spectrum of mepacrine effects on pores and channels would suggest that mepacrine interactions with the CPE pore are not specific. It deserves mention that mepacrine derivatives have been shown to be much more active than the native drug against the pore activity of C2 and lethal toxins (44), further supporting the possibility (mentioned earlier) that more efficacious drugs in the mepacrine family may be identified.

To our knowledge, this study reports the first evidence that mepacrine can protect relevant host cells from the activity of a toxin that acts by forming pores in the eukaryotic plasma membrane. Future studies are planned to evaluate whether mepacrine might also protect host cells against other toxins that contribute to disease by forming pores in plasma membranes.

## MATERIALS AND METHODS

### *C. perfringens* enterotoxin and rCPE_D48A_.

CPE was purified to homogeneity from *C. perfringens* strain NCTC 8238 (ATCC 12916), as described previously for strain NCTC 8239 (45). The recombinant CPE variant, rCPE_D48_, was prepared and shown in a previous study to bind but not oligomerize or form pores (14). This recombinant CPE species was purified to near homogeneity, as described previously (14).

### Caco-2 cell culture.

In a humidified incubator, authenticated Caco-2 cells were grown in Eagle’s minimal essential medium (Lonza) containing 10% fetal bovine serum (Sigma), 1% nonessential amino acids (Sigma), 2 mM glutamine (Sigma), and 100 μg/ml of penicillin-streptomycin (Fisher Scientific) at 37°C with 5% atmospheric CO_2_.

### Characterization of CPE-induced Caco-2 cell cytotoxicity in the presence of mepacrine.

Caco-2 cells were grown to confluence in 6-well plates and washed once with warm (37°C) Hanks’ balanced salt solution containing Ca^2+^ and Mg^2+^ (Mediatech). These Caco-2 cells were then either (i) preincubated with mepacrine (at concentrations between 0 and 600 μM) (Caymen Chemical) in HBSS for 30 min at 37°C prior to a 30- to 240-min treatment at 37°C with both CPE (1 μg/ml) and the same concentration of mepacrine as used for the precubation, or (ii) treated directly with CPE (1 μg/ml, unless otherwise noted) in 37°C HBSS containing mepacrine (between 0 and 600 μM) and incubated at 37°C for 30 to 240 min. Following this incubation, death of Caco-2 cells was measured by using a cytotoxicity detection kit (Roche; based on lactate dehydrogenase [LDH] release) according to the manufacturer’s instructions. Cytotoxicity is expressed as the percentage of maximal LDH release after correction for spontaneous (background) LDH release.

### CPE exposure to mepacrine and analysis of potential posttreatment effects.

Mepacrine (600 μM) was incubated with CPE (1 μg/ml) for 1 h at 37°C. Following incubation, the CPE was dialyzed overnight to remove free mepacrine. The following morning, CPE alone or mepacrine-treated CPE was added to Caco-2 cells for 1 h at 37°C, and LDH release was measured.

A second experiment was performed in which mepacrine (600 μM) was incubated with CPE (25 μg/ml) for 1 h at 37°C. Following incubation, the mepacrine-treated CPE or untreated CPE was placed over a PD-10 column. After elution of CPE, dilutions of mepacrine-treated CPE or untreated CPE were incubated with Caco-2 cells for 1 h at 37°C, and LDH release was measured as described above. A small fraction was saved to measure fluorescence at 488 nm to determine if mepacrine had associated with CPE.

### Assessment of mepacrine effects on CPE large complex formation in Caco-2 cells.

Confluent Caco-2 cells grown in six-well plates were treated for 60 min with 1 μg/ml of CPE in 37°C HBSS containing mepacrine (0 to 600 μM). Following this treatment, cells were gently removed from each plastic culture dish with a rubber cell scraper and collected by centrifugation for 5 min. After a gentle wash with HBSS, the pelleted cells were resuspended in HBSS containing Benzonase and incubated for 10 min at room temperature. Laemmli SDS-PAGE reducing sample buffer (46) was added to the collected cells to solubilize the cellular membranes containing CPE large complex. Samples were then loaded onto 6% (for Western blotting) or 10% (to ensure equal protein loading) acrylamide gels containing SDS. After electrophoresis, separated proteins were transferred onto nitrocellulose membranes for Western blotting or stained with Coomassie G-250 to ensure equivalent protein loading. Recent evidence suggests that various pathological conditions can influence the amounts of proteins commonly used for loading analysis and that total protein analysis is a more accurate means of ensuring equal loading than immunodetection of a single protein (47). For this purpose, total protein analysis by Coomassie staining was used as a loading control in these studies. Blots were blocked with 5% milk in phosphate-buffered saline with 0.2% Tween 20 (PBS-T) and incubated with rabbit anti-CPE antibody (11) overnight at 4°C. Following three washes with PBS-T, blots were incubated with secondary goat anti-rabbit IgG horseradish peroxidase-conjugated antibody in 5% milk in PBS-T, washed with PBS-T, and developed with SuperSignal West Pico chemiluminescent substrate (Thermo Fisher).

### Characterization of mepacrine effects on CPE binding to Caco-2 cells.

Caco-2 cells in confluent monolayers were preincubated at 4°C for 30 min with 5 μg/ml of CPE in HBSS containing mepacrine (0 to 600 μM) to allow CPE binding but not oligomerization or pore formation (11). Following this preincubation, cells were washed three times with 4°C HBSS. Prewarmed 37°C HBSS was then added, and cells were incubated at 37°C for 60 min before LDH release and large complexes were measured, as described above.

In an additional experiment, 2.5 μg of rCPE_D48A_, purified as previously described (14), was incubated with Caco-2 cells for 30 min at 37°C in the presence and absence of mepacrine. Following three washes with warm HBSS, cells were harvested, lysed, and processed for Western blotting. Bound rCPE_D48A_ was detected as described above.

### Characterization of mepacrine effects on CPE postbinding activity.

Confluent monolayers of Caco-2 cells grown in six-well plates were preincubated at 4°C for 30 min with 5 μg/ml of CPE in HBSS. Following this preincubation, cells were washed three times with 4°C HBSS. Prewarmed 37°C HBSS with mepacrine (0 to 600 μM) was then added and cells were incubated at 37°C for 60 min before LDH release and large complex formation were measured as described above.

### Analysis of CPE-induced cytotoxicity and large complex formation in Caco-2 cells after pretreatment with mepacrine was followed by CPE treatment in the absence of mepacrine.

Confluent cultures of Caco-2 cells grown in six-well plates were preincubated at 37°C for 30 min with mepacrine (0 to 600 μM) in HBSS. Following that pretreatment, cells were washed three times with prewarmed 37°C HBSS. After washing, CPE in prewarmed HBSS was added at a concentration of 1 μg/ml and incubated at 37°C for 1 h. After that CPE treatment, LDH release and large complex formation were measured as described above.

### Detection of the release of CPE species in supernatants from CPE-treated Caco-2 cell cultures.

Caco-2 cell monolayers grown to confluence were treated at 4°C for 30 min with 5 μg/ml of CPE in HBSS to allow toxin binding to the cells. Unbound toxin was removed by three washes with 4°C HBSS before warm HBSS was added with or without 600 μM mepacrine for 1 h at 37°C. Cells were then collected with gentle scraping and centrifuged at 2,500 × *g* for 5 min. Supernatants were gently removed and then centrifuged for 30 min at 10,000 × *g* to collect LMVs. The LMV-depleted supernatants were ultracentrifuged (90 min at 100,000 × *g*) to remove SEVs from the supernatants. Equivalent volumes of the resulting fractions (cells, LMVs, SEVs, or final supernatants) were then analyzed by Western blotting for CPE monomer and large complex formation as described above. ImageJ analysis was performed on 3 separate Western blots to determine the fold increase in free, monomeric CPE present in the final supernatants. Pan-cadherin was used to measure relative vesicle release (for both LMVs and SEVs) in mepacrine-treated or untreated cells, as previously described (30).

### ^86^Rb release.

Caco-2 cells grown to confluence in a 24-well plate were incubated with 4 μCi/well of ^86^Rb (PerkinElmer) at 4°C for 3 h in Caco-2 growth medium. Radiolabeled cells were washed twice with 37°C HBSS buffer before HBSS or HBSS with CPE (2.5 μg/ml) was added to cells for 2.5 min at 37°C, which allowed CH-1 pore formation in the CPE-treated cells. Following this incubation, cells were washed twice with 37°C HBSS to remove unbound CPE. Either HBSS or HBSS with 600 μM mepacrine was then added to the cells and the mixtures were incubated for 15 min at 37°C. The CPE-induced percentage of maximal ^86^Rb release was then calculated as previously described (15, 36, 37).

### Statistical analyses.

One-way analysis of variance (ANOVA) with Dunnett’s *post hoc* test were used to compare data sets against the control mepacrine-untreated cells in experiments with greater than 2 groups, and Student’s unpaired *t* test was used when comparing only 2 sets of data.
